# Modeling present and future climate risk of dengue outbreak, a case study in New Caledonia

**DOI:** 10.1186/s12940-022-00829-z

**Published:** 2022-01-20

**Authors:** Noé Ochida, Morgan Mangeas, Myrielle Dupont-Rouzeyrol, Cyril Dutheil, Carole Forfait, Alexandre Peltier, Elodie Descloux, Christophe Menkes

**Affiliations:** 1 UMR ENTROPIE (IRD, Université de la Réunion, CNRS, Ifremer, Université de la Nouvelle-Calédonie), Nouméa, New Caledonia; 2 URE-Dengue et Arboviroses, Institut Pasteur de Nouvelle-Calédonie, Pasteur Network, Nouméa, New Caledonia; 3 Department of Physical Oceanography and Instrumentation, Leibniz Institute for Baltic Sea Research, Warnemünde, Rostock, Germany; 4 Direction des Affaires Sanitaires et Sociales, Nouméa, New Caledonia; 5 Météo France, Nouméa, New Caledonia; 6 Service de Médecine interne, Centre Hospitalier Territorial Gaston-Bourret, 988935 Dumbea-Sur-Mer, New Caledonia

**Keywords:** dengue, disease outbreaks, effective reproduction number, climate change, prediction

## Abstract

**Background:**

Dengue dynamics result from the complex interactions between the virus, the host and the vector, all being under the influence of the environment. Several studies explored the link between weather and dengue dynamics and some investigated the impact of climate change on these dynamics. Most attempted to predict incidence rate at a country scale or assess the environmental suitability at a global or regional scale. Here, we propose a new approach which consists in modeling the risk of dengue outbreak at a local scale according to climate conditions and study the evolution of this risk taking climate change into account. We apply this approach in New Caledonia, where high quality data are available.

**Methods:**

We used a statistical estimation of the effective reproduction number (*R*_*t*_) based on case counts to create a categorical target variable : epidemic week/non-epidemic week. A machine learning classifier has been trained using relevant climate indicators in order to estimate the probability for a week to be epidemic under current climate data and this probability was then estimated under climate change scenarios.

**Results:**

Weekly probability of dengue outbreak was best predicted with the number of days when maximal temperature exceeded 30.8°C and the mean of daily precipitation over 80 and 60 days prior to the predicted week respectively. According to scenario RCP8.5, climate will allow dengue outbreak every year in New Caledonia if the epidemiological and entomological contexts remain the same.

**Conclusion:**

We identified locally relevant climatic factor driving dengue outbreaks in New Caledonia and assessed the inter-annual and seasonal risk of dengue outbreak under different climate change scenarios up to the year 2100. We introduced a new modeling approach to estimate the risk of dengue outbreak depending on climate conditions. This approach is easily reproducible in other countries provided that reliable epidemiological and climate data are available.

**Supplementary Information:**

The online version contains supplementary material available at 10.1186/s12940-022-00829-z.

## Background

Dengue is the most common arboviral disease in the World. Approximately half the world’s population is considered at risk and 390 millions persons are infected each year [1]. Climate is a major determinant of mosquito-borne disease and dengue is no exception. Climate influences dengue ecology by affecting vector dynamics, virus replication and vector/human interactions. For a review of association between climate and dengue transmission see [2].

In parallel, climate change is expected to be one of the main driver of environmental change at the global scale in the future [3]. Given its large distribution around the globe and its link with climate, dengue is expected to be greatly affected by the ongoing global changes in earth’s climate. Abounding research studies have investigated the associations between climate and dengue transmission and several used associations between climate and dengue to make predictions about the future of dengue based on climate projections [4–8]. However most studies have focused on predicting dengue incidence in the future and seldom studies have projected probability of dengue outbreak or risk of dengue outbreak for a specific location in the face of climate change [9–12]. The latter relied on mechanistic models that use simplified mathematical formulation of biological processes to make predictions. In these models, important parameters for dengue dynamics could be missing or rigid equations may not capture the influence of local context on dengue ecology for specific locations. In contrary with machine learning algorithms, given their empirical nature, biological processes involved in the relation between features and the target variable are implicit.

In this present study we introduced a complete method to forecast the current weekly probability of dengue outbreak for a specific location (e.g. New Caledonia) based on climate variability and estimate the inter-annual and seasonal risk of dengue outbreak during the next century facing climate change. We used New Caledonia as a working example which is a South Pacific island which face recurrent seasonal dengue outbreaks. Dengue profile in New Caledonia and its association with environmental variables has been documented in previous studies for the past 50 years thanks to a long record of quality surveillance data [13].

A complete process is presented, from data collection, to data pre-processing, model designing, features selection and application to future climate projections. We address a number of methodological issues such as the importance to define an adapted target variable and the processing of potential non-linearities between epidemiological data and climate variables.

## Methods

### Study area

This study takes place in New Caledonia, a French overseas autonomous territory located in the southwest Pacific between 19°S and 23°S about 1,200km east of Australia and 1,500 km north of New Zealand. New Caledonia is subject to both tropical and temperate influences depending on the season [14]. There are two main seasons. The hot season is centered on the first quarter of the year. The tropical influence is predominant. Precipitation is abundant and average temperatures are high although extremes are limited by the maritime influence and the trade winds. The cool season, from June to September, where the weather is generally dry and cool with relatively low minimum temperatures in some areas. The population was estimated in January 2020 to be 271,407. New Caledonia has a multicultural and inclusive population constructed around Melanesian, Oceanian, Asian and European cultures. Approximately half of inhabitants are concentrated in Noumea, the main city and its greater urban area [14].

### Epidemiological data

Demographic data come from general population census of New Caledonia made by the Institut de la Statistique et des Etudes Economiques (ISEE). Times series of population have been made by linear interpolation of the general population census: 1969, 1976, 1983, 1989, 1996, 2004, 2009, 2014 and 2019 [15]. In New Caledonia, dengue is a notifiable disease. Monthly number of dengue cases from 1973 to 2019 have been retrieved from the Public Health Authorities of New Caledonia. Dengue cases are defined as clinical or confirmed. A clinical case is an evocative dengue case without diagnosis. A confirmed cased has been confirmed by direct detection of dengue virus by reverse-transcriptase polymerase chain reaction (RT-PCR using a pandengue technique) and/or serological assay (IgM detection by indirect immunofluorescence or ELISA) [16].

### Meteorological data

Daily rainfall (RR) and maximal temperature (TX) were measured by a weather station of Météo-France from 1970 to 2020 at Faubourg Blanchot, Nouméa. A moving average on TX and RR was computed in order to create climate indicators. The time windows of the moving average varied from 50 days to 80 days preceding each current week w for TX and 30 days to 70 days for RR. Additionally, number of days where these variables exceeded a panel of given threshold (e.g, number of days where TX exceeded 32°C) were computed as Descloux et al., [16] indicated that such thresholds on temperatures and rainfall were most pertinent for predicting outbreaks. We thus obtain a large panel of climate indicators (Table 1). Before computation, RR was log-transformed to normalize its distribution for better predictions.

**Table 1:** Description of the meteorological data and climate indicators generated

Variables	Description (units)	Source	Mean (SD)	Median	5th - 95th percentile
TX	Maximum daily air temperature (°C)	Time serie of maximum daily air temperature for the period 1965-2019 retrieved from the the Météo-France weather station in Noumea	26.47 (2.92)	26.5	22.0 - 31.4
RR	Daily precipitation (mm)	Time serie of daily precipitations for the period 1965-2019 retrieved from the Météo-France weather station in Noumea	2.97 (10.08)	0.00	0.00 - 15.34
NOD_TX_GT_D_Q	Number of days (D) with maximal air temperature exceeding its given quantile (Q) with associated probability *p*, preceding the predicted week *D* ∈ {50,55,60, …, 75, 80} *p* ∈ {0.900,0.905, …, 0.985,0.990}	Computed from TX	-		
NOD_TX_LT_D_Q	Number of days (D) with maximal air temperature below its given quantile (Q) with associated probability *p*, preceding the predicted week. *D* ∈ {50,55,60, …, 75, 80} *p* ∈ {0.100,0.105, …, 0.295,0.300}	Computed from TX	-		
NOD_RR_GT_D_Q	Number of days (D) with daily precipitation exceeding its given quantile (Q) with associated probability *p* preceding the predicted week *D* ∈ {30,35,40, …, 65, 70} *p* ∈ {0.800,0.805, …, 0.945,0.950}	Computed from RR	-		
NOD_RR_LT_D_Q	Number of days (D) with daily precipitation below its given quantile (Q) with associated probability *p* preceding the predicted week *D* ∈ {30,35,40, …, 65, 70} *p* ∈ {0.500,0.505, …, 0.795,0.800}	Computed from RR	-		
MEAN_TX_D	Average maximal air temperature during a period of D days preceding the predicted week	Computed from TX	-		
MEAN_RR_D	Logarithm of the average daily precipitation during a period of D days preceding the predicted week	Computed from RR	-		

### Climate change scenarios data

To obtain projections of temperature and rainfall under different global warming scenarios, we retrieved historical (1970-2004) and projections (2005-2100) of daily maximum temperature and rainfall simulated by eight coupled ocean-atmosphere models from the 5th Phase of the Coupled Model Intercomparison Project – Assessment Report 4 (CMIP5 – AR4) experiments [17]. The eight selected models were “CanESM2”, “CNRM-CM5”, “inmcm4”, “IPSL-CM5A-MR”, IPSL-CM5B-LR”, “MPI-ESM-LR”, MRI-CGCM3” and “NorESM1-M”. They were selected based on their capacity to reproduce the observed climate in the South Pacific [18]. Three scenarios of emission (greenhouse gases and aerosols) – referred to as “Representative Concentration Pathways” (RCPs) were chosen: the RCP8.5 for a high emission scenario, RCP4.5 for a midrange mitigation emission scenario and RCP2.6 for a low emission scenario. These data are thereafter referred to as "historical" covering 1971-2004 and “projections” (RCP2.6, RCP4.5 and RCP8.5) covering 2005-2100. For each model, we selected time-series from the spatial point closest to Noumea. A statistical downscaling based on a quantile-quantile correction was also applied to correct the distribution of the modeled time series to fit the distribution observed time series over the historical period [19]. This correction is then applied to projections to correct accordingly their distributions as in [19]. That allowed avoiding a part of model biases while keeping their climate change trends.

### Risk of dengue outbreaks assessment

The risk of dengue outbreak was estimated through the effective reproduction number *R*_*t*_ defined as the number of secondary infections caused by a primary case at time *t*. If *R*_*t*_ > 1 the number of cases increases with time, *R*_*t*_ must be *< 1* for an outbreak to decline. *R*_*t*_ was estimated according to a method proposed by Wallinga & Teunis [20] and computed thank to the “R0” package implemented by Obadia et al. in R [21]. This method allowed to transform a time series of cases in a time series of estimated values of *R*_*t*_ and require as input the “Generation Time” distribution (i.e. the time lag between infection in a primary case and a secondary case). Based on the extrinsic incubation period of the virus within the mosquito (2 to 15 days at 30°C) – and intrinsic incubation period of the virus within human (3 to 9 days) [22], we assumed the generation time distribution was ‘lognormal’ with a mean of 14 days and a standard deviation of 7 days. *R*_*t*_ was computed only when incidence rates (per 100,000 inhabitants) where superior to the 80th percentile and set to 0 otherwise in order to avoid high values of reproduction number with low circulation of the virus.

In the end, based on the *R*_*t*_ computed, the two classes of our target variable was defined as following: an epidemic week is a week where *R*_*t*_ > 1 and a non-epidemic week is a week where *R*_*t*_ < 1.

### Model building

To avoid multi-collinearity between explanatory variables and consider the possible existence of complex non-linear links between the explanatory variables and the response variable, we chose to use Support Vector Machines classifier (SVM) to model the weekly risk of dengue outbreak [23].

In training phase, to compensate for imbalance in the dataset, 175 epidemic weeks versus 2269 non epidemic weeks, we adjusted the class distribution by a random oversampling of epidemic weeks. To determine the model with the best prediction performance (i.e. “best model”), a model was created for each combination of one or more climate indicators as inputs (i.e. explanatory variables). Then after training, the models performance were compared on a test set in a 5 fold Leave-Time-Out Cross Validation (LTO-CV) [24]. The method consists in dividing the time series of data in 5 continuous equal parts (i.e. time segment) to account for the time nature of the data. The model is trained on four parts and test on the fifth. Estimated probability of dengue outbreak for the current week (between 0 and 1) were compared to the actual state of the week (1 if epidemic, 0 otherwise) then the performances were compared in terms of Brier score.

We further studied the uncertainties associated to the covariates importance in the best model selected by LTO-CV. Toward that end, we computed an importance score for each variable as follows: once the model was fitted, the importance score was calculated using 1000 random permutations of the covariate of interest in the original dataset thus creating 1000 corrupted datasets. The loss of predictive performance of the model on the corrupted dataset was quantified by the ratio of that model Brier score calculated on the corrupted dataset to the Brier score of the original best model.

The marginal effect of each variable of the best model on the probability of a week being epidemic was characterized by Partial Dependence plots (PDP). The plot shows for each value of the variable of interest the marginalized predicted probabilities of dengue outbreak over the distribution of the other variables. The mean of predicted probabilities of dengue outbreak is represented by the plain line and standard deviation by the shaded area. At the top of the graph is represented for each variable, their distribution in the original dataset.

The software R and packages, e1071 [25], were used for the simulations. In the end, the model provides a probability for each week w, that *R*_*t*_ > 1 (i.e. weekly probability of dengue outbreak).

### Forecasting inter- annual and seasonal dengue outbreak risk variability

The evolution of the risk of dengue outbreak under climate change scenario is explored by feeding the “best model” with climate indicators up to 2100 from the projections : RCP2.6, RCP4.5 and RCP8.5. A year was considered epidemic if for at least one week during that year, the probability was greater than a set threshold. Thresholds were chosen so that the predictions of the model fed with observed climate data and the model fed with modeled climate data are consistent. Thresholds were fixed to 0.6 for the model fed with the input from the observed climate data and 0.8 for the model fed with the modeled climate data. Then the inter-annual risk variability was assessed for the entire period 1973-2100 for the three warming scenarios. To highlight long-term trend, inter-annual risk was computed as a central moving average of 11 years where the value for each year is 1 if an outbreak is observed, 0 otherwise.

The seasonal risk variability was assessed by averaging the weekly probability of dengue outbreak for each of the 52 weeks of the year in 4 different time frames 1973-2004, 2020-2040, 2050-2070 and 2080-2100. Confidence intervals were also estimated based on the variance of the eight selected climate models forecasts.

#### Results

**Fig 1:**
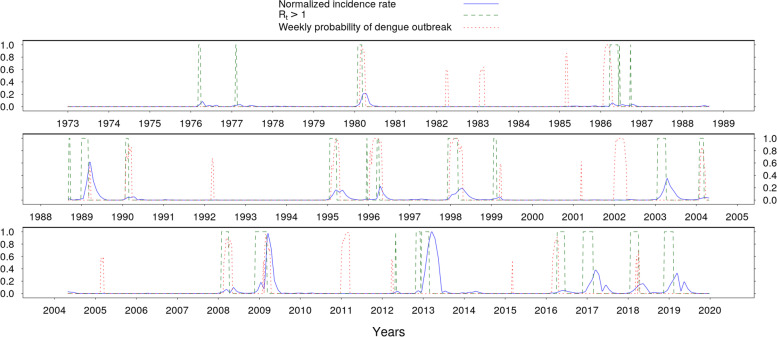
Climate based prediction of weekly dengue outbreak risk during 1973-2020 in New Caledonia.

The best model for estimating the weekly probability of dengue outbreak from 1973 to 2020 used the number of days with maximal temperature exceeding 30.8°C during a period of 80 days preceding the predicted week and the logarithm of the mean daily precipitation during a period of 60 days preceding the predicted week as input. Adding additional variables in the LTO-CV selection did not improve the model in terms of Brier score. Looking at our model, most of the periods conducive to epidemic growth were detected in the contemporary period (i.e. 1973-2020) (Fig. 1).

The dashed red line depicts the model probability of dengue outbreak each week (*w*) according to the number of days with maximal temperature exceeding 30.8°C during a period of 80 days preceding w and the logarithm of mean daily precipitation during a period of 60 days preceding *w*. Normalized weekly incidence rate is in solid blue line. Periods prone to dengue outbreaks (*R*_*t*_ > 1) are in dashed green line.

**Fig 2:**
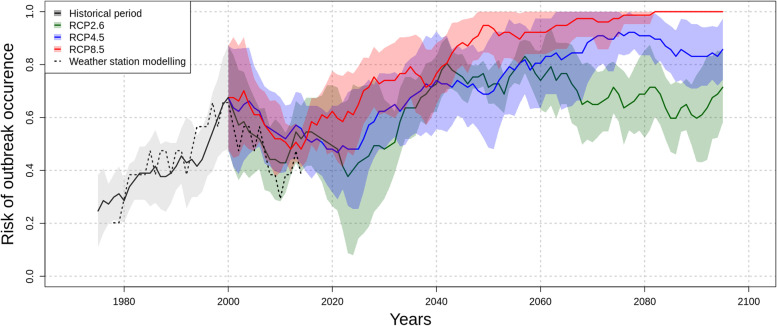
Evolution of the inter-annual risk of dengue outbreak until 2100 per RCP scenarios

The model failed to detect “double outbreaks” (i.e. spread of the two years) where the model predicted high risk either the first or the second year. Feeding the model with CMIP5 projections of temperature and rainfall under the global warming scenarios: RCP2.6, RCP4.5 and RCP8.5, we were able to estimate the smoothed yearly risk of dengue outbreak up to 2100 (Fig. 2).

Plain lines denotes the yearly mean of dengue outbreak risk estimated by the model based on the 8 selected CMIP5 models for the historical period and the three scenarios. Black lines depicts the historical period, green line depicts the low greenhouse gas emission scenario (RCP2.6), blue line depicts the medium emission scenario (RCP4.5) and red line depicts the high emission scenario (RCP8.5). The risk is computed as a central moving average (11 years) of the epidemic year time series; with epidemic year (i.e. a year where at least one week was estimated epidemic) coded as 1 and non epidemic year coded as 0. For each lines, the corresponding colored region depicts the confidence interval (±1 standard deviation of the 8 selected models). Processed in a similar way, the dashed black line denotes the risk estimated based on the weather station records during the contemporary period (1973-2020).

Over the CMIP5 projections historical period (i.e. before the divergence of the three RCP scenarios), there is concordance in the estimated risk of dengue outbreak between inputs with the weather station climate data and the modeled climate data. This reassured us about the validity of using such data. Note that the risk estimated with the modeled climate data appeared smoother because it is average over 8 models. When the scenarios diverge, the estimated risk of dengue outbreak from the model fed with the weather station climate data seems to follow the trend of the model fed with the RCP2.6 climate data. Based on the CMIP5 projections and the data from the weather station, the general trend is a decrease in risk after 2000, then around 2020 it increases again and reach an asymptote in 2100 around 0.7 for RCP2.6, 0.8 for RCP4.5 (i.e. 4 outbreaks out of 5 years) and 1 for RCP8.5 (i.e. one outbreak every year).

**Fig 3:**
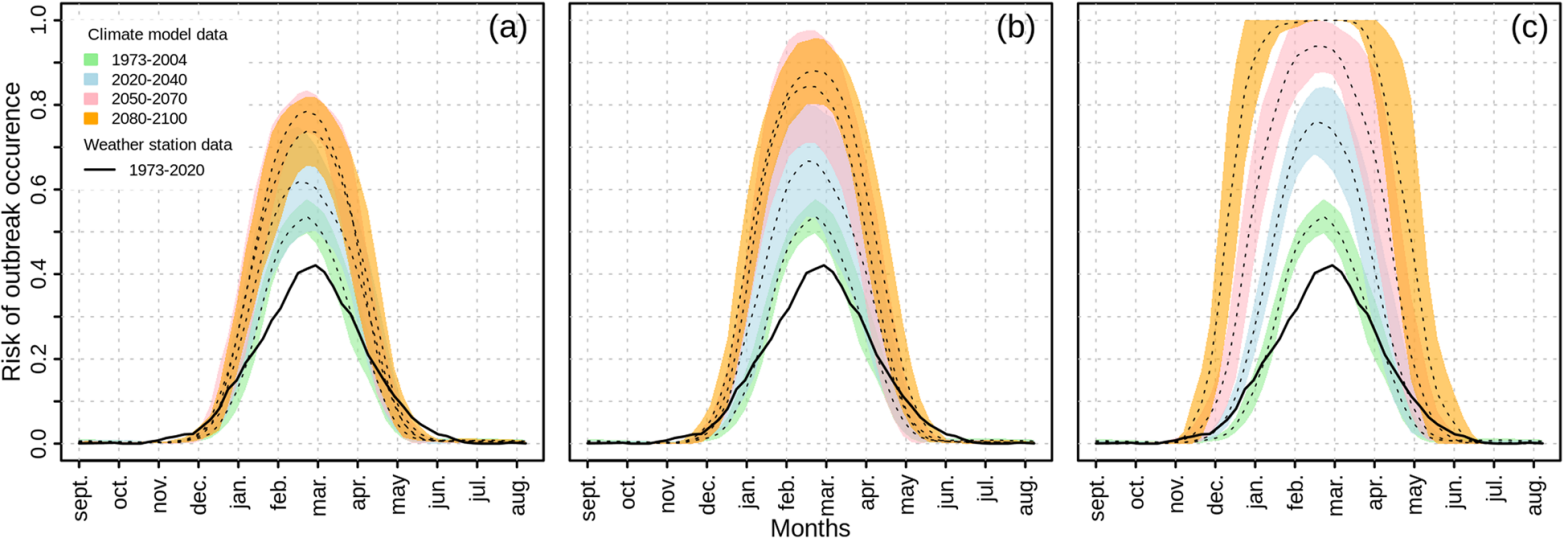
Evolution of the seasonal risk of dengue outbreak until 2100 per RCP scenarios

After investigating the inter-annual risk of dengue outbreak, we looked at the evolution of the profile of the seasonal risk of dengue outbreak. This risk computed as the average probability of a week being epidemic over 4 distinct time frames is presented Fig. 3.

From left to right, each frame depicts the seasonal dengue outbreak risk according to **(a)** the low greenhouse gas emission scenario (RCP2.6), **(b)** the medium emission scenario (RCP4.5) and **(c)** the high emission scenario (RCP8.5) respectively. Dotted lines are the mean of the weekly risk of dengue outbreak estimated by the model based on the 8 selected CMIP5 models and colored region denotes the confidence interval around that mean (±1 standard deviation of the 8 selected models) averaged for the period 1973-2004 in green, 2020-2040 in light blue, 2050-2070 in pink and 2080-2100 in orange.

Despite similar dynamic there is an upward trend in the risk estimated with the CMIP5 climate data compare to the risk estimated with the weather station climate data. According to all CMIP5 scenarios, the seasonal risk gradually increase for each periods until 2100 but seasonality remains well marked with no risk of dengue outbreak in winter. For the scenarios RCP2.6 and RCP4.5 the risk of dengue outbreak reaches a plateau in 2050.

## Discussion

Results showed that climate change may increase the inter-annual risk of dengue outbreak in New Caledonia despite the fact that seasonality will remain well marked with zero risk of epidemic emergence during winter. According to RCP8.5, climate will permit outbreaks to emerge every year in New Caledonia from 2080. Assuming that epidemiological and vector contexts will remain the same, this will make dengue endemic in New Caledonia. Nevertheless it is likely that dengue epidemiology will change if outbreaks gain in frequency and amplitude. More people will develop immunity and the size of the susceptible population will become one of the main driver in the epidemic dynamic. Also, New Caledonia currently attempt to reduce vector transmission capacity by introducing *Wolbachia* vector control method which may change drastically the dengue dynamic. In this study, we just claim that the climate conditions will be more suitable in the future for triggering dengue outbreaks.

This future inter-annual and seasonal risk was estimated using a SVM algorithm capable of estimating the probability of a week conducive to epidemic growth with climate indicators. The features selected by LTO-CV that best predicted the weekly probability of dengue outbreak were the number of days with maximal temperature exceeding 30.8°C during a period of 80 days prior the predicted week and the logarithm of the mean daily precipitation during a period of 60 days prior the predicted week. A table of the best models selected by LTO-CV is presented in supplementary material (Table S1).

**Fig. 4:**
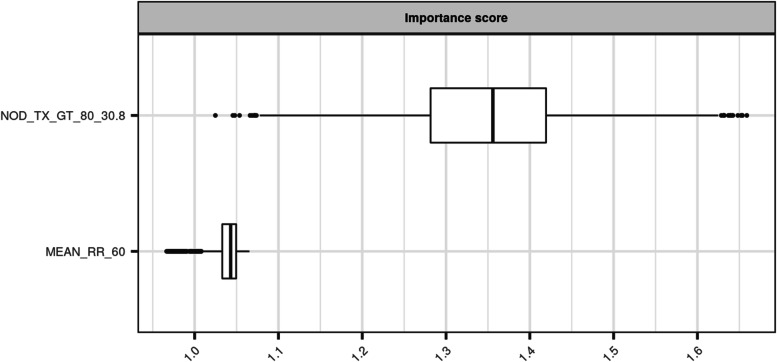
Importance score of the selected variables in the SVM model

Inspecting the variables selected by the best model in more details, we can see from their importance scores that the most important variable to predict an epidemic week is the number of days with maximal temperature exceeding 30.8°C during a period of 80 days prior the predicted week. The impact of the second variable on the model predictive abilities is shown to be much weaker (Fig. 4).

This score compares the increase in terms of Brier score caused by 1000 permutations of the variable of interest in the original dataset thus creating 1000 corrupted datasets. Results are presented in the form of a boxplot (*BrierScore*_*corrupteddataset*_/*Brierscore*_*originaldataset*_). NOD_TX_GT_80_30.8 : number of days with maximal temperature exceeding 30.8°C during a period of 80 days preceding the predicted week and MEAN_RR_60 : logarithm of the mean daily precipitation during a period of 60 days preceding the predicted week.

**Fig. 5:**
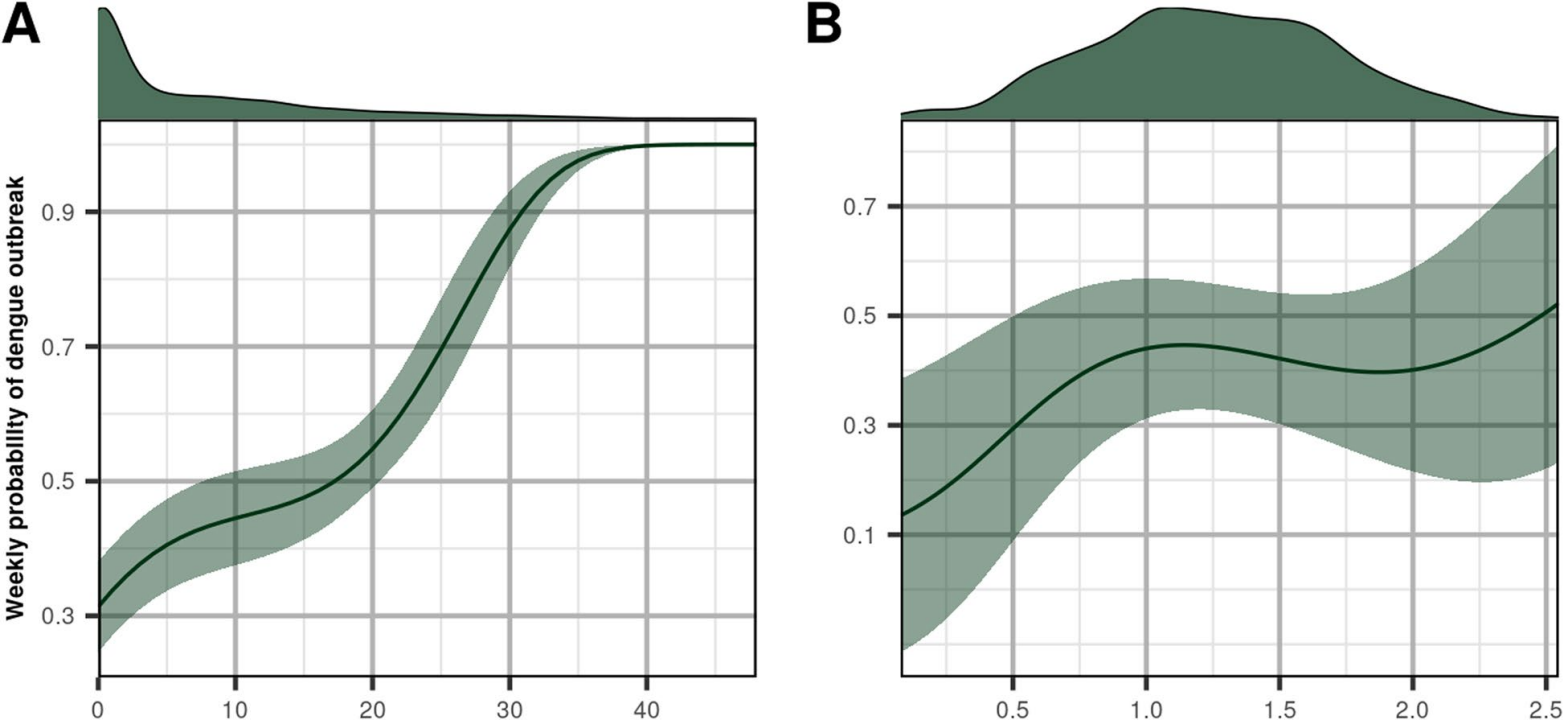
Partial dependence of the probability of dengue outbreak to the selected variables in the SVM model

The two selected features exhibit mostly monotonic non-linear relationship with the weekly probability of dengue outbreak as it is revealed by the partial dependence plots (Fig. 5). According to the model, the weekly probability of dengue outbreak is maximal when the number of days with maximal temperature exceeding 30.8°C during a period of 80 days preceding the predicted week is roughly greater than 35 days (i.e, approximately 1 out of 2 days during previous 80 days shows temperature greater than 30.8°C). In that temperature regime, the model predicts a 100% chance of an epidemic week. For the precipitation variable, the weekly probability of dengue outbreak globally increases with increasing mean daily precipitation during a period of 60 days preceding the predicted week especially when passing from a dry regime (no precipitation) to a wetter regime leading to a ~50% chance for an epidemic week at most.

The plot shows for each value of the variable of interest the marginalized predicted probabilities of dengue outbreak over the distribution of the other variables. The mean of predicted probabilities of dengue outbreak is represented by the plain line and standard deviation by the shaded area. At the top of the graph is represented for each variable, their distribution in the original dataset.

A: NOD_TX_GT_80_30.8 : number of days with maximal temperature exceeding 30.8°C during a period of 80 days preceding the predicted week; B: MEAN_RR_60 : logarithm of the mean daily precipitation during a period of 60 days preceding the predicted week.

The choice of the SVM algorithm for predictions was motivated by the fact that it does not make a priori on the nature of the relationship between the explanatory variables and the response variable. In the present study a non-linear link was found between temperature, precipitation and dengue outbreaks which is consistent with the literature [2, 26]. For example Colon-Gonzàlez et al. showed highly non linear effects of weather on dengue incidence in Mexico with a peak effect of temperature around 32°C which is coherent with our temperature feature [27]. We note that on the contemporary period (1973-2020), our model failed to detect “double outbreaks”. For these outbreaks, immunity may play a bigger role in the epidemic dynamic, diminishing the role of climatic factors. In fact, new serotype introductions, genotypic displacements or serotypic replacements may have played a bigger role in these outbreaks [13].

A key element in our study is the use of the effective reproduction number *R*_*t*_ to spot periods conducive to epidemic growth or not (respectively *R*_*t*_ > 1 or *R*_*t*_ < 1) and use it as a categorical target variable (i.e. epidemic week/ non epidemic week). Thus size of outbreaks (e.g. incidence) are not considered. Size of outbreaks may be more sensitive to non-climatic factors such as the size of the susceptible population or the behavior of the human host population. In New -Caledonia, as mention in [13] the size of the susceptible population does not seem to be a limiting factor in triggering an outbreak since we have observed outbreaks with the same serotype for several consecutive years in the past. Concerning our statistical estimation of *R*_*t*_, by directly specifying the “Generation Time” distribution, time lags between favorable climate condition and epidemic growth are considered. Shifting the focus from dengue incidence to periods conducive to epidemic growth may lead to more robust assessment of the risk of dengue outbreak.

According to all models in our CMIP5 projections, temperature shows a robust increase until 2100 (Fig. S1). The intensity of this increase depends on the scenario considered. For instance in RCP8.5, a mean increase of 0,82°C is found in 2020-2040 and 2,98°C in 2080-2100 in comparison to the historical period 1973-2004. In RCP2.6, a mean increase of 0.68°C is found in 2020-2040 and 0.82°C in 2080-2100 in comparison to the historical period 1973-2004. In parallel, rainfall does not show marked changes in the future whatever the scenario considered. Thus temperature may be considered as the solely driver of the increased annual and seasonal risk of dengue outbreaks in the future in New Caledonia.

It might be argued that the used of CMIP5 models is inappropriate because they have a poor horizontal resolution (~100-200km). Indeed CMIP5 models are not adapted to simulate the climate of a mountain island such as New Caledonia. Nevertheless, the amplitude of temperature changes simulated by CMIP5 models is robust and perform well in comparison to an atmospheric regional model at the island scale [28]. In contrary, Dutheil et al. showed that projections of precipitations simulated by CMIP5 models may not be reliable [29]. These authors showed a possible strong reduction of precipitation over New Caledonia at the end of the 21^st^ century for RCP8.5 scenario. This is prompting further work to re-assess the risk of dengue outbreaks in New Caledonia facing climate change with high resolution climate data from regional climate projections.

In conclusion, we introduced a method to assess the weekly probability of dengue outbreak for a specific location and project this risk under climate change scenario. With this method we demonstrated that climate change may increase the inter-annual risk of dengue outbreak in New Caledonia despite the fact that seasonality will remain well marked with zero risk of dengue outbreak during winter.

## Supplementary Information

**Additional file 1: Figure S1.** Evolution of maximal temperatures according to different RCP scenarios up to the year 2100.

**Additional file 2: TableS1.** Top 50 best predictive models selected by Leave Time Out - Cross Validation (LTO-CV) according to their Brier score.

## Data Availability

Dengue case counts data that support the findings of this study are available from New Caledonia Public Health Services but restrictions apply to the availability of these data, which were used under convention for the current study, and so are not publicly available. Data are however available from the authors upon reasonable request and with permission of New Caledonia Public Health Services. Meteorological data that support the findings of this study are available from New Caledonia Meteorological Department (Météo France) but restrictions apply to the availability of these data, which were used under licence for the current study, and so are not publicly available. Data are however available from the authors upon reasonable request and with permission of Météo France. Projections of future climate data are available upon request.

## Abbreviations

CMIP: Coupled Model Intercomparison Project
ELISA: Enzyme-Linked Immunosorbent Assay
ISEE: Institut des Statistiques et des Etudes Economiques
LTO-CV: Leave Time Out Cross Validation
RCP: Representative Concentration Pathways
RR: Daily rainfall
RT-PCR: Reverse Transcriptase Polymerase Chain Reaction
TX: Maximal temperature
SVM: Support Vector Machine
