# Supplementary figures and images for: Modeling present and future climate risk of dengue outbreak, a case study in New Caledonia
Source: Environ Health. 2022 Jan 20;21:20. doi: 10.1186/s12940-022-00829-z (PMC8772089; doi:10.1186/s12940-022-00829-z)

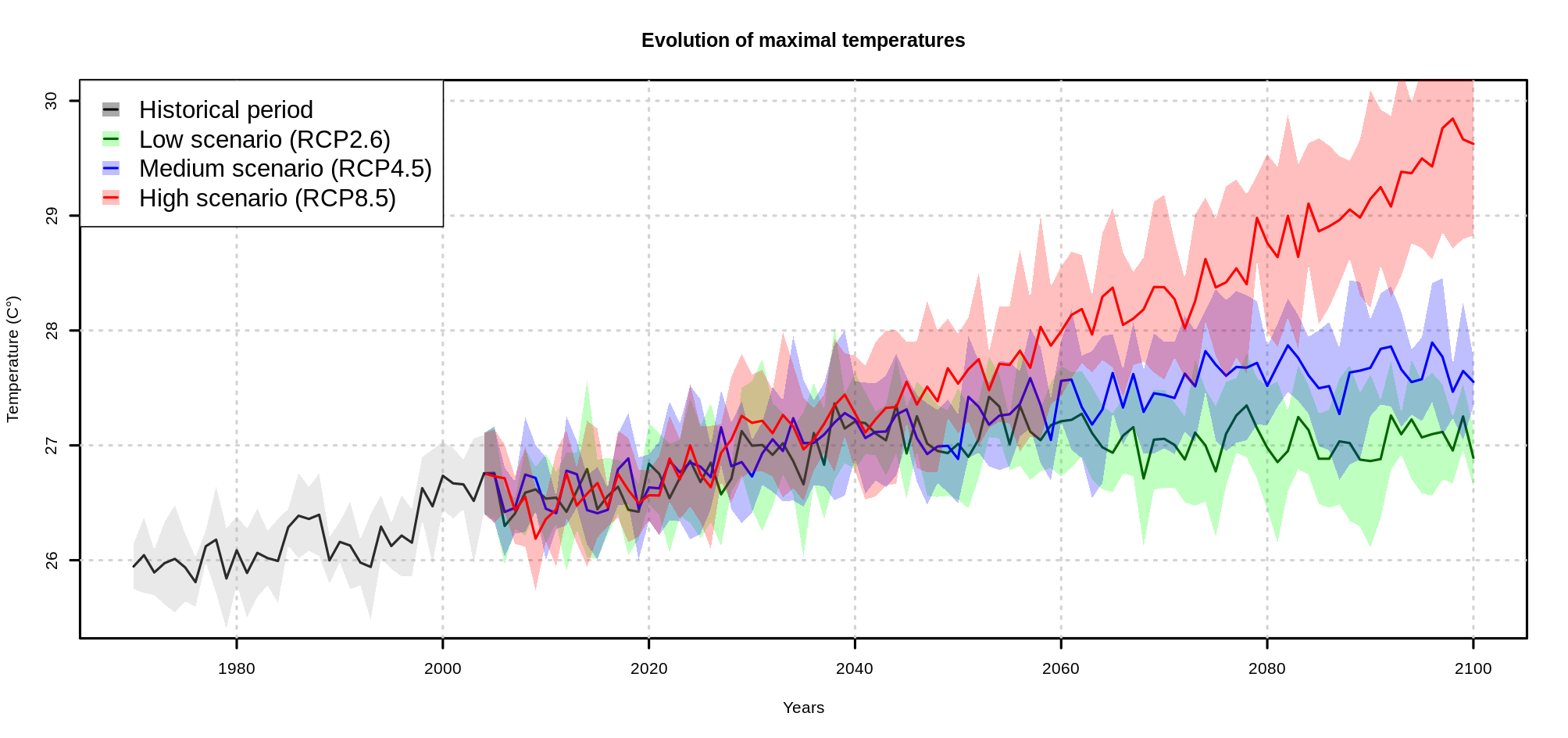

Supplement: Supplementary file 1 — Additional file 1: Figure S1. Evolution of maximal temperatures according to different RCP scenarios up to the year 2100. [file 12940_2022_829_MOESM1_ESM.tiff]
